# Variant Allele Characterization in STR Markers Using Next-Generation Sequencing

**DOI:** 10.3390/genes17060617

**Published:** 2026-05-29

**Authors:** Lauren E. Mullen, Carolyn R. Steffen, Katherine B. Gettings, Kevin M. Kiesler, Peter M. Vallone

**Affiliations:** National Institute of Standards and Technology, 100 Bureau Drive, Gaithersburg, MD 20899, USA; lauren.mullen@nist.gov (L.E.M.); becky.steffen@nist.gov (C.R.S.); kevin.kiesler@nist.gov (K.M.K.); peter.vallone@nist.gov (P.M.V.)

**Keywords:** variant allele, next-generation sequencing, Sanger sequencing, null allele, off-ladder allele

## Abstract

**Background/Objectives**: Traditionally, Sanger sequencing was used to characterize reference materials and confirm discordant allele calls from different STR typing kits at the National Institute of Standards and Technology (NIST). Sequencing can also identify genomic variations within polymerase chain reaction (PCR) amplicons containing STRs, particularly variants that result in null alleles and alleles that do not migrate within allele sizing bins provided by kit manufacturers. **Methods**: Sanger methods are low-throughput, time- and labor-intensive, and require additional procedures for analysis of heterozygous alleles. To address these limitations, a quicker, more straightforward protocol that uses next-generation sequencing (NGS) was developed. **Results**: This research provides the criteria used to individually sequence thirty-five autosomal STR loci, with PCR primer locations chosen to increase amplicon length and maximize the likelihood of detecting variants in the flanking region. The list of targeted sequences, associated primers, and chromosomal coordinates is also included. **Conclusions**: By applying NGS technology to forensic samples containing variant alleles, additional information can be obtained about their molecular basis, and this information can be published and shared across the forensic community. The development of this protocol can increase awareness and encourage the integration of NGS technology into forensic laboratories to improve forensic DNA typing for human identification.

## 1. Introduction

Forensic DNA typing is a comparative technique used by investigators to identify individuals by analyzing unique patterns within their genetic material. This method focuses on typing short tandem repeats (STRs), which are short repeating sequences of DNA at specific locations on a chromosome [1]. Because the number of repeats at any given site varies from person to person, forensic scientists can measure these lengths to create a DNA profile. The measured PCR amplicon lengths are compared to an allelic ladder, which is a mixture of DNA fragments containing the most common known alleles (repeat lengths) for the assignment of an allele to an unknown sample [1].

Variant short tandem repeat (STR) alleles do not conform to the expected allele sizes that a kit vendor considers when developing an allelic ladder (or the bins/panels settings within the analysis software). Most of the variant alleles observed are microvariants and/or “off-ladder alleles,” in which an insertion/deletion mutation (indel) is present in the STR or the region between the STR sequence and the PCR priming site, known as the flanking region, resulting in an allele size different from the allelic ladder fragments [2]. For these variants, laboratories can extrapolate the allele designation based on the neighboring allelic ladder fragment sizes or designate that allele as greater or less than the closest ladder allele. A larger indel can cause an allele to migrate between two loci within the same dye channel in an electropherogram [3]. In extreme cases, the off-ladder allele from one locus can migrate into the region of a different locus in the electropherogram [4], creating potential for misinterpretation. In addition to off-ladder alleles, a single-nucleotide polymorphism or an indel in a PCR primer binding region can lead to reduced or no amplification, also called a null allele, in which the allele appears at a lower peak height or drops out altogether [2].

As part of the forensic genetics projects at the NIST, samples that were STR typed by traditional capillary electrophoresis (CE) methods and demonstrated off-ladder or imbalanced/null allele calls at a locus were sequenced for that specific locus to identify the cause of the observed CE electropherogram result. Over the years, candidate samples were compiled primarily through discordances identified in the NIST U.S. population samples when genotyped with multiple commercial CE STR typing kits, during the pre-release testing of new CE STR typing kits, and in the development of in-house multiplexes [2,5,6].

**Figure 1:**
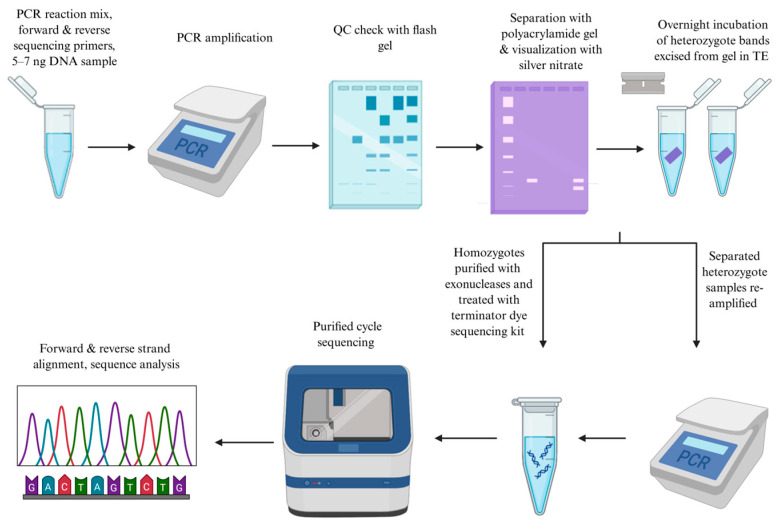
Sanger sequencing workflow for variant allele characterization. Created in BioRender. Mullen, L. (2026) https://BioRender.com/aix20du.

**Figure 2:**
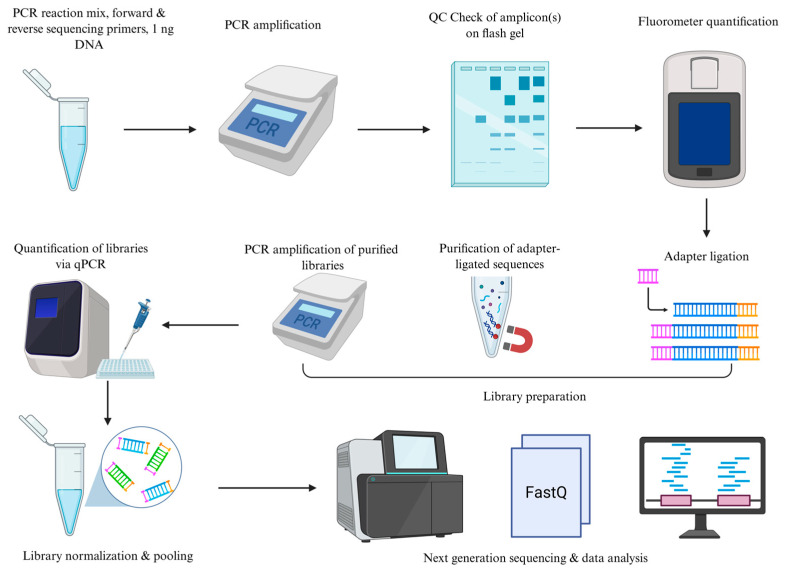
Next-generation sequencing workflow for variant allele characterization. Created in BioRender. Mullen, L. (2026) https://BioRender.com/gey8oxt.

Historically, this effort relied on Sanger sequencing methods. For heterozygous loci, the alleles must be physically separated, and the subsequent excised bands incubated overnight in TE^−4^ buffer prior to Sanger sequencing to avoid “phasing” issues that complicate and obscure the interpretation of the sequencing electropherogram. Otherwise, the sequences would be obscured by a mixture of signals from the two alleles [2]. The Sanger sequencing method, as implemented for variant allele sequencing at the NIST, required five to seven nanograms of extracted DNA template for PCR amplification and sufficient visualization on an acrylamide gel, as opposed to a single nanogram being sufficient for an NGS-based method, making the latter a more favorable option (See Figure 1 and Figure 2 for Sanger and NGS workflow diagrams). The nature of NGS methods presents a more efficient technology for this application, as all reads originate from a single DNA template molecule and are distinctly observable; thus, the physical separation of heterozygous alleles is not required.

Here, we describe a method for sequencing one or more samples containing a suspected variant at a targeted STR locus included in commercial forensic STR typing kits using NGS-based techniques. The initial proof of this method was performed for thirty-five commonly used autosomal STR loci.

## 2. Materials and Methods

### 2.1. Samples

Component C from the NIST Standard Reference Material (SRM) 2391d PCR-Based DNA Profiling Standard (human DNA extracts) at a concentration of 1 ng/µL was used as the DNA template during the initial phases of protocol development when the PCR parameters were being modified [7]. After the preliminary phase, NIST U.S. population samples with known variants were used to evaluate the protocol’s efficacy under the chosen parameters.

### 2.2. Primer Design Criteria

With the eventual goal of identifying sequence variations within the flanking regions of commercial amplification kits, the primer locations were chosen to span a broad range of the flanking regions on both ends of the target region. Because the exact primer locations of the targeted loci in commercial amplification kits are proprietary information, we instead designed primer locations that would result in amplicon sizes larger than those present in commercial kits, to the extent possible without exceeding approximately 500 base pairs (bp) in amplicon size. To facilitate primer design that encompasses the commercial primer sites, amplicon length comparisons were made across twenty-seven commercial PCR amplification kits from Promega Corporation (Madison, WI, USA), Thermo Fisher Scientific (Waltham, MA, USA), and QIAGEN (Germantown, MD, USA). Tables of these amplicon length comparisons can be found in Supplementary Tables S1–S3.

Initially, thirty-eight autosomal STR loci found across various commercial STR amplification kits were targeted for monoplex assay design (See Supplementary Tables S1–S3). Primer design was performed by first obtaining sequences from the human genome reference assembly, GRCh38.p14, that contained the STR region and extended flanking regions on each end of the repeat. The sequences were input into Primer3, and the primer-selection conditions were set as described in [8,9,10,11]. These conditions included the desired amplicon length range, GC content, primer length range, and primer annealing temperature (Tm). Initially, the conditions were limited to a small range; if no primers were identified within that range, the range was expanded. The GC content percentage was initially set to be between 45% and 55%, and the annealing temperature was set to be between 59 °C and 61 °C. The default setting of 18 to 23 bp was used for the primer size range. The amplicon length range was determined by identifying the minimum length required to exceed the amplicon lengths targeted in commercial amplification kits without exceeding 500 bp from end to end. To simplify the protocol development where possible, the primers were designed so that the subsequent PCR amplification could be performed using one set of thermal cycler parameters regardless of the locus being targeted, wherein a 60 °C Tm was chosen as the optimal temperature, with a maximum variation of ±2 °C when primers resulting in the required amplicon length range were unable to be generated at the optimal Tm. Once primer sequences that fell sufficiently within the chosen parameters were selected, they were input into the National Institutes of Health’s NCBI Primer Blast tool to verify chromosomal location and assess non-specific binding [12]. These chromosome coordinates were then entered into gnomAD v4.0.0 using GRCh38.p14 as a reference to identify reported single-nucleotide polymorphisms (SNPs) within the primer binding sites [13]. If a SNP in the primer binding site occurred at a frequency greater than 0.1% in any of the population groups recorded in gnomAD v4.0.0, alternative primers were identified and assessed using the same process described above. Previously designed primers for Sanger sequencing that did not meet the chosen criteria were redesigned for NGS. A complete list of primers, sequences, and their chromosomal coordinates (GRCh38.p14) for the successfully targeted and sequenced loci is provided in Supplementary Table S4.

### 2.3. PCR Mix and Amplification Parameters

Primers were synthesized by Eurofins Scientific (Luxembourg, Belgium) and diluted to a working concentration of 10 µM. Platinum II Taq Hot-Start DNA Polymerase enzyme (Thermo Fisher Scientific) was used because of its optimization for a 60 °C Tm. The master mix was prepared without primers, as one set of primers targeting a single locus was added individually to each amplification reaction. Multiple PCR amplifications were performed for each targeted locus, starting at 30 cycles and increasing the number of cycles until dark bands with minimal smearing were observed using the Lonza FlashGel System (Walkersville, MD, USA) to visualize the resulting PCR products. It was determined that 35 cycles yielded a sufficient quality and quantity of PCR product at each locus for downstream sequencing. The final PCR component volumes and amplification parameters are provided in Supplementary Protocol S1. Three loci (D2S1338, D3S3045, and D5S2500) amplified poorly and require primer redesign; these three loci were not carried forward to library preparation and sequencing. PCR products for the remaining thirty-five loci were quantified using the Qubit Fluorometer (Thermo Fisher Scientific) and the manufacturer’s Qubit dsDNA High Sensitivity Assay Kit protocol to ensure at least two nanograms per microliter of PCR product was present prior to library preparation [14].

### 2.4. Adapter Ligation and Library Cleanup

Library preparation was performed using the QIAGEN QIAseq 1-Step Library Amplification Kit and the QIAseq 1-Step Amplicon Library UDI-A Kit (96) [15]. This included one-step end repair and ligation of dual-index adapters from the QIAseq UDI Y-Adapter Kit A plate, followed by several wash steps using AMPure XP Beads (Beckman Coulter, Brea, CA, USA). Amplification of the purified library was performed using the manufacturer’s recommended protocol to target 50 ng of PCR product; however, all reagent and template volumes were halved for the one-step adapter ligation, resulting in a 25 µL total reaction volume rather than the 50 µL described in the manufacturer’s protocol. In early testing, this was compared with results obtained with the full reaction volume, and no differences in library quality or quantity were observed. Manufacturer-recommended reagent volumes for all other steps were used for the remainder of the library preparation.

### 2.5. Quantitation of Libraries

Libraries were quantified using the QIAGEN QIAseq Library Quant Assay Kit following the Real-Time PCR for QIAseq Library Quant Assay Kit for Ion Torrent or Illumina protocol [16]. All steps were performed following the manufacturer’s recommended protocol and using primers for the Illumina platform. Instrument parameters were selected according to the manufacturer’s recommendations for qPCR using the Applied Biosystems 7500 Real-Time PCR System (Waltham, MA, USA).

### 2.6. Library Pooling

Libraries were prepared for sequencing using the MiSeq System Denature and Dilute Library Guide Protocol A: Standard Normalization Method, and initially diluted to 4 nM. Equal volumes of the 4 nM library and 0.2 N NaOH were combined for denaturation, and then the subsequent denatured library was diluted further, resulting in a 12 pM loading concentration [17]. This protocol was also used to dilute and denature the PhiX control to 12 pM. A 10% spike-in of 12 pM PhiX Control v3 (Illumina, San Diego, CA, USA) was combined with the prepared libraries for sequencing.

### 2.7. Sequencing

The MiSeq Reagent Nano Kit v2 (500-cycles) (Illumina) was used to sequence the samples on a MiSeq FGx Sequencing System in Research Use Only (RUO) mode. This kit was chosen for its cost-effectiveness in sequencing a small number of samples, making it ideal for sequencing a suspected variant. Furthermore, the targeted amplicons in most commercial PCR amplification kits range from approximately 100 to 500 bp, making a 500-cycle reagent kit more likely to include the region of interest than a lower-cycle-number kit. Although the Nano Kit yields up to two million paired reads, compared with at least fifteen million with other MiSeq reagent kits, this was proven sufficient for accurate variant allele characterization.

### 2.8. Verification of Sequences and Data Analysis

Initial sequencing was performed to confirm that sufficiently high-quality sequence data could be generated at each locus using the selected primers and protocol conditions. SRM 2391d Component C was amplified in monoplex PCRs (each reaction containing only one primer set), each monoplex PCR product was labeled with a unique index combination (barcode) during library preparation, and all barcoded monoplex sample libraries were combined into a single library pool for sequencing. For analysis, paired-end run fastq.gz files from the sequencer were imported into Geneious Prime v.2024.0.5 (Dotmatics Inc., Boston, MA, USA) for sequence analysis. Both read 1 (R1) and read 2 (R2) at each locus were imported simultaneously to generate a single paired read file. These locus-specific sequences were then aligned with the corresponding GRCh38.p14 reference sequence (see Supplementary Table S4 for coordinates used to obtain reference sequences). This same data analysis process was performed during later testing of samples containing known variants, using GRCh38.p14 as the reference sequence. Once aligned, sequence differences between the reference and the sample were evaluated.

### 2.9. Nucleotide Diversity

When a small number of loci are targeted in a sequencing run, and particularly when the loci exhibit highly unequal distributions of nucleotide bases, all four bases may be unevenly represented in each cycle of sequencing. If a base is underrepresented in one cycle and this continues to occur over the course of multiple cycles, the lack of fluorescence may cause clusters to be rejected by the software filter. The resultant poor sequence quality and significant fluorescent signal imbalance in a single channel can become problematic [18]. This may be reflected in a cluster density below the recommended target range, measured in thousands of clusters per square millimeter (K/mm^2^). This was observed in the later phases of research once all other aspects of the protocol were optimized and samples were sequenced individually or in small groups, targeting only a few markers at a time to identify variants. Specifically, this was observed after a sequencing run targeting only two loci, both of which were highly unbalanced in their base distributions. This run had a cluster density of 690 K/mm^2^, which is below the recommended range of 1000 to 1200 K/mm^2^ for sequencing with the Illumina MiSeq Reagent Nano Kit v2, and a passing filter value of 8%, indicating a significant number of base-calling errors [18]. Because this protocol was developed to target only a locus containing a suspected variant, this lack of nucleotide diversity became a recurring issue for many locus combinations. This can be addressed by increasing the PhiX control concentration in the pooled libraries to produce a more equal distribution of bases, as recommended by the manufacturer [18]. However, the volume and concentration of PhiX control would require optimization for each sequencing run. As an alternative, PCR primers from five STR loci with a nearly equal distribution of all four bases were combined for the multiplex amplification of D22S1045, D13S317, D16S539, D8S1179, and TPOX. Going forward, when variants are characterized via sequencing, this five-plex can be amplified with a control sample (e.g., NIST SRM 2391d) and processed alongside the variant samples. This allows the PhiX spike-in to remain consistent while ensuring the nucleotide diversity required for high-quality sequencing data.

## 3. Results

### 3.1. SRM 2391d Component C Sequencing Results

**Figure 3:**
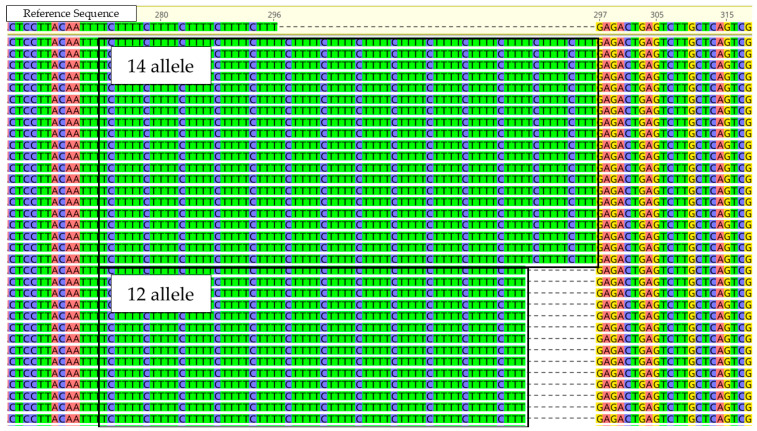
Sequence data of SRM 2391d Component C at locus Penta E. The GRCh38.p14 reference sequence contains a 5 allele, whereas SRM 2391d Component C has a genotype of 12,14. The 5 [TCTTT] repeats of the reference sequence are shown at the top of the figure. Black outlining delineates the 14 [TCTTT] repeats from the 12 [TCTTT] repeats.

Initial analysis of sequence data was performed using previously established STR sequence data for SRM 2391d Component C to evaluate the reliability of this methodology for characterizing variant alleles [7]. An example of sequence data results is shown in Figure 3 at the Penta E locus. In this example, the GRCh38.p14 reference sequence used to align the sample sequences is a “5 allele” (an allele containing 5 repeats), and SRM 2391d Component C is known to be heterozygous at this locus, with a genotype of 12,14. This analysis of 2391d Component C produced the expected results for the thirty-five loci successfully sequenced, confirming that the desired region within the genome was properly targeted and that the expected repeat motifs, as well as flanking regions, were sequenced.

### 3.2. Variant Allele Sequence Data

**Figure 4:**
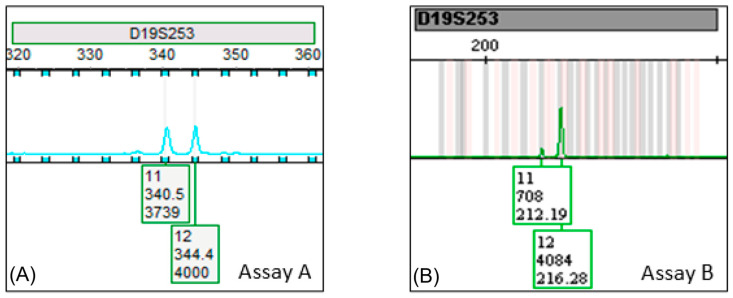
Comparison of results at D19S253 from the same sample genotyped with two different CE-based assays. Assay A resulted in a well-balanced 11,12 genotype (**A**), and assay B resulted in an imbalanced (17.3% PHR) 11,12 genotype (**B**), where the 11 allele would likely be interpreted as an n-1 stutter. NOTE: Different instruments and software were used to genotype and analyze results from assays A and B due to one having been designed for 8-dye chemistry and the other for 5-dye chemistry.

A suspected null allele at locus D19S253 was initially detected when comparing the genotype of sample ZT80369 from the NIST population sample set using two different CE-based assays (referred to herein as assay A and assay B for simplicity). As shown in Figure 4, the genotype generated using assay A was a well-balanced 11,12 at this locus, whereas the genotype from assay B appeared as a homozygous 12,12 (4084 relative fluorescent units or RFUs), with a small peak observed in the 11 allelic ladder bin (708 RFUs). Given the peak height ratio of 17.3% between the 11 and the 12 allele, this peak would likely be interpreted as an n-1 stutter peak, meaning that the 11 allele observed in assay A either completely dropped out or amplified poorly with assay B.

**Figure 5:**
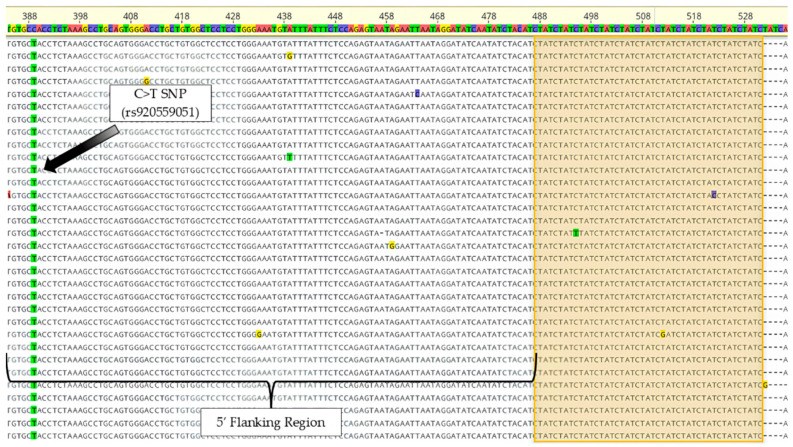
Sample ZT80369 sequence data of a variant allele at locus D19S253; The reference sequence shown in the yellow box at the top of the figure contains 12 [TATC] repeats, and the sample sequences below contain 11 [TATC] repeats highlighted in orange. The four dashes immediately after the repeat region indicate the one repeat difference between the reference and the sample. A C>T SNP (rs920559051) is observed in the 5′ flanking region of the sample, approximately 100 bp from the repeat region, in which all the T bases are highlighted in green. This SNP was determined to be the likely cause of the poorly amplified 11 allele obtained with assay B.

**Figure 6:**
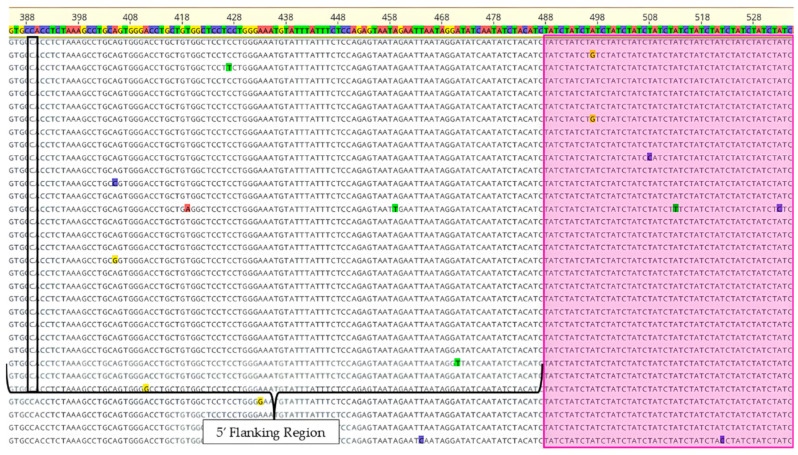
Sample ZT80369 sequence data of 12 allele at locus D19S253; The reference sequence in the yellow box at the top of the figure contains 12 [TATC] repeats, and the sample sequences below also contain 12 [TATC] repeats highlighted in pink. The C>T SNP observed in the sample sequences of the 11 allele (see Figure 5) is not present in the 12 allele sequences.

Through analysis of targeted sequencing data of sample ZT80369 at locus D19S253, a C>T SNP was located in the 5′ flanking region of the 11 allele sequence. It was not observed in the flanking sequence of the 12 allele. This SNP is likely to be within the primer-binding region of assay B, resulting in the attenuated amplification of the 11 allele when amplified with this assay. This determination was supported by the sequencing data in Figure 5, which shows a subset of the 11 allele sequences of the sample in comparison to a reference sequence from GRCh38.p14 and the location of the C>T SNP (rs920559051) in the 5′ flanking region. Sequence data of the 12 allele, which does not contain the SNP, is shown in Figure 6.

## 4. Discussion and Conclusions

Sequencing the region surrounding an STR is important in identifying the cause of microvariant, “off-ladder”, or null alleles observed as unexpected or missing peaks in traditional CE analysis of STRs. Variability in primer placements, with exact locations being proprietary and varying amplicon sizes across commercial CE genotyping kits, makes it important to investigate the flanking regions of STRs at the sequence level to characterize the causes of differences observed between kits. By developing an NGS-based protocol, variant allele sequencing can be performed more efficiently than with Sanger sequencing for thirty-five autosomal STR loci (see Supplementary Table S4). The clonal nature of NGS methods eliminated the need to separate and isolate heterozygous alleles prior to sequencing.

As previously mentioned in Section 2.7, the MiSeq Reagent Nano Kit v2 (500-cycles) was selected for its cost-effectiveness in comparison to other MiSeq reagent kits. A maximum of two million reads is sufficient for variant allele sequencing performed at the NIST, as in most cases, only a few select samples are sequenced at a time. The coverage will vary depending on how many samples are sequenced in a single run, which can be up to 96 with this chemistry. The variant sample shown in Figure 5 and Figure 6 had over 85,000 paired-end reads, which confidently identified the SNP in the flanking region that caused the variant allele. Artifacts such as minus-one stutter are similar to those observed in traditional CE typing, which was approximately 8% in the discussed example.

During the development of this NGS method, challenges arose in designing primers with uniform amplification parameters for each monoplexed locus. In many cases, multiple primer pairs were tested for a locus before primers were found that enabled the successful sequencing of that marker. PCR parameters may need to be modified to amplify additional loci not addressed in this work if it is not possible to design primers conforming to the conditions discussed in Section 2.2. Of the samples containing suspected variants sequenced since the protocol’s implementation, all have led to the successful identification of the variant. However, this may not always be the case. Because the primer regions in commercial kits are proprietary and unknown, the SNP causing a variant may not be detected if the kit’s primers target a region outside the sequenced region at a locus. In scenarios where the SNP’s specific location is unknown, primers can be redesigned to extend further across the 5′ and 3′ flanks. For example, two complementary primer sets can be utilized: one extending past the 5′ boundary to the end of the 3′ repeat region, and another extending past the 3′ boundary to the end of the 5′ repeat region. Areas for future investigation include determining the minimum number of markers required for a single sequencing run to provide sufficient nucleotide diversity, thereby minimizing fluorescent signal imbalance across the four bases and producing high-quality data. For sequencing a small number of STR loci, the PhiX spike-in concentration can be optimized to mitigate sequence diversity needs without compromising the depth of coverage of the target samples. Expansion of this project will continue in the future with the addition of primers targeting X- and Y-STRs found in commercial STR amplification kits, as well as the redesign of primers for the loci D2S1338, D3S3045, and D5S2500.

## Data Availability

The original contributions presented in this study are included in the article/Supplementary Material. Further inquiries can be directed to the corresponding author.

## Supplementary Materials

The following supporting information can be downloaded at: https://www.mdpi.com/article/10.3390/genes17060617/s1, Supplementary Protocol S1: Variant Allele Sequencing; Table S1. Amplicon length ranges for Promega Corporation (Madison, WI) PCR amplification kits. Chromosome locations were obtained from the human genome reference assembly, GRCh38.p14. The allele call of the GRCh38 sequence at each locus is indicated in bolded, red font in column B. Table S2. Amplicon length ranges for Thermo Fisher Scientific (Waltham, MA), PCR amplification kits. Chromosome locations were obtained from the human genome reference assembly, GRCh38.p14. The allele call of the GRCh38 sequence at each locus is indicated in bolded, red font in column B. Table S3. Amplicon length ranges for QIAGEN (Germantown, MD, USA) PCR amplification kits. Chromosome locations were obtained from the human genome reference assembly, GRCh38.p14. The allele call of the GRCh38 sequence at each locus is indicated in bolded, red font in column B. Table S4. Forward and reverse primer sequences and locations of thirty-five forensically relevant STR markers for variant allele characterization with NGS. Chromosome locations were obtained from the human genome reference assembly, GRCh38.p14. The grayed-out rows denote the loci that require primer redesign. Table S5. PCR Amplification Reaction Mix. Table S6. Amplification Parameters.
