# Supplementary material for: Variant Allele Characterization in STR Markers Using Next-Generation Sequencing
Source: Genes (Basel). 2026 May 29;17(6):617. doi: 10.3390/genes17060617 (PMC13299351; doi:10.3390/genes17060617)
Supplement: Supplementary file 1 [file genes-17-00617-s001.zip › Supplementary Protocol S1.pdf]

## Supplementary Protocol S1: Variant Allele Sequencing

Reagent List:

- Invitrogen Platinum™ II Taq Hot-Start DNA Polymerase (5X Platinum II PCR Buffer, Platinum II Taq Hot-Start DNA Polymerase, and Platinum GC Enhancer) (catalog no. 14966001)
- dNTPs
- Bovine Serum Albumin (BSA)
- Primer oligomers
- QIAGEN QIAseq 1-Step Amplicon Library Kit (catalog no. 180419)
- QIAGEN QIAseq 1Step Amplicon Lib UDI-A Kit (96) (catalog no. 180419)
- QIAGEN QIAseq Library Quant Assay Kit (catalog no. 333314)
- Illumina MiSeq Reagent Nano Kit v2 (500-cycles) (catalog no. MS-103-1003)

### A. PCR Amplification Sample Preparation

1. Prepare master mix containing PCR buffer, dNTPs, DNA polymerase, GC enhancer, BSA, and deionized water according to Table 1.
   a. If additional sequencing template is needed for nucleotide diversity (i.e., when sequencing a small number of samples with highly unequal distributions of nucleotide bases), a separate multiplex (e.g., D22S1045, D13S317, D16S539, D8S1179, and TPOX – see section 2.9 of this paper) can be amplified on a separate sample (e.g., 2800 M) and added to the flow cell.
2. Vortex and briefly centrifuge the master mix and pipette the appropriate volume into 0.2 mL tube(s).
3. Pipette primers (at 10 µM concentration) targeting the locus of interest and DNA into the appropriate sample tube.
4. Cap tubes and briefly centrifuge.
5. Program the thermocycler according to the parameters outlined in Table 2.

**Table S5:** PCR Amplification Reaction Mix

| PCR mix component | Volume per reaction |
|---|---|
| 5X Platinum™ II PCR Buffer | 4.0 µL |
| dNTPs (10 mM) | 0.4 µL |
| Platinum™ II Taq Hot-Start DNA Polymerase | 0.16 µL |
| GC enhancer | 4.0 µL |
| Bovine Serum Albumin (BSA) (3.2 mg/mL) | 2.5 µL |
| Deionized water | 5.94 µL |
| DNA template at 1.0 ng/µL | 1.0 µL |
| Forward primer at 10 µM | 1.0 µL |
| Reverse primer at 10 µM | 1.0 µL |
| Total | 20.0 µL |

**Table S6:** Amplification Parameters

| Amplification Parameters: | | |
|---|---|---|
| 1 X | 94 °C | 2 min |
| 35 X | 94 °C | 15 sec |
| | 60 °C | 30 sec |
| | 68 °C | 96 sec |
| 1 X | 4 °C | Hold |

Ramp Rate: 6 °C/Sec

Note: During protocol development, thermal cycling was performed on the Applied Biosystems™ ProFlex™ PCR System. Other thermocyclers have not been tested.

**B. Adapter Ligation and Library Cleanup**

1. Follow library preparation steps according to the QIAGEN QIAseq 1-Step Library Amplification Kit protocol and the QIAseq 1-Step Amplicon Library UDI-A Kit (96) protocol.
    a. Half or full volume reactions can be used for the amplification of purified libraries.

**C. Quantitation of Libraries**

1. To quantify purified libraries, follow the steps according to the QIAGEN QIAseq Library Quant Assay Kit, following the Real-Time PCR for QIAseq Library Quant Assay Kit for Ion Torrent or Illumina protocol.
2. Use the measured concentrations to dilute each library to 4 nM prior to library pooling in section D.

**D. Library Pooling and Sequencing**

1. Combine 5 µL of each 4 nM library and prepare for sequencing according to the MiSeq System Denature and Dilute Library Guide Protocol A: Standard Normalization Method for 4 nM libraries.
2. Denature 4 nM libraries and dilute for a final loading concentration of 12 pM.
3. Follow the MiSeq protocol to denature a 4 nM PhiX control library and dilute to 12 pM.
4. Add 60 µL of the 12 pM PhiX control library to 540 µL of the 12 pM library for a 10 % PhiX spike-in.
5. Load the 600 µL library into the MiSeq Nano reagent cartridge for sequencing on a MiSeq instrument in RUO mode.

Note: During protocol development, sequencing was performed on the MiSeq FGx sequencer in RUO mode with a MiSeq Reagent Nano Kit v2 (500 cycles). Other sequencers and reagent kits have not been tested.
